# An overview of methods to address distinct research questions on environmental mixtures: an application to persistent organic pollutants and leukocyte telomere length

**DOI:** 10.1186/s12940-019-0515-1

**Published:** 2019-08-28

**Authors:** Elizabeth A. Gibson, Yanelli Nunez, Ahlam Abuawad, Ami R. Zota, Stefano Renzetti, Katrina L. Devick, Chris Gennings, Jeff Goldsmith, Brent A. Coull, Marianthi-Anna Kioumourtzoglou

**Affiliations:** 1 Department of Environmental Health Sciences, Columbia University Mailman School of Public Health, New York, NY USA; 2 Department of Environmental and Occupational Health, George Washington University Milken Institute School of Public Health, Washington, DC USA; 3 Occupational Health, Department of Molecular and Translational Medicine, University of Brescia, Brescia, Italy; 4 Department of Biostatistics, Harvard T.H. Chan School of Public Health, Boston, MA USA; 5 Department of Environmental Medicine and Public Health, Icahn School of Medicine at Mount Sinai, New York, NY USA; 6 Department of Biostatistics, Columbia University Mailman School of Public Health, New York, NY USA

**Keywords:** Environmental mixtures, Chemical mixtures, Multi-pollutant, Dimension reduction, Variable selection

## Abstract

**Background:**

Numerous methods exist to analyze complex environmental mixtures in health studies. As an illustration of the different uses of mixture methods, we employed methods geared toward distinct research questions concerning persistent organic chemicals (POPs) as a mixture and leukocyte telomere length (LTL) as an outcome.

**Methods:**

With information on 18 POPs and LTL among 1,003 U.S. adults (NHANES, 2001–2002), we used unsupervised methods including clustering to identify profiles of similarly exposed participants, and Principal Component Analysis (PCA) and Exploratory Factor Analysis (EFA) to identify common exposure patterns. We also employed supervised learning techniques, including penalized, weighted quantile sum (WQS), and Bayesian kernel machine (BKMR) regressions, to identify potentially toxic agents, and characterize nonlinear associations, interactions, and the overall mixture effect.

**Results:**

Clustering separated participants into high, medium, and low POP exposure groups; longer log-LTL was found among those with high exposure. The first PCA component represented overall POP exposure and was positively associated with log-LTL. Two EFA factors, one representing furans and the other PCBs 126 and 118, were positively associated with log-LTL. Penalized regression methods selected three congeners in common (PCB 126, PCB 118, and furan 2,3,4,7,8-pncdf) as potentially toxic agents. WQS found a positive overall effect of the POP mixture and identified six POPs as potentially toxic agents (furans 1,2,3,4,6,7,8-hxcdf, 2,3,4,7,8-pncdf, and 1,2,3,6,7,8-hxcdf, and PCBs 99, 126, 169). BKMR found a positive linear association with furan 2,3,4,7,8-pncdf, suggestive evidence of linear associations with PCBs 126 and 169, and a positive overall effect of the mixture, but no interactions among congeners.

**Conclusions:**

Using different methods, we identified patterns of POP exposure, potentially toxic agents, the absence of interaction, and estimated the overall mixture effect. These applications and results may serve as a guide for mixture method selection based on specific research questions.

**Electronic supplementary material:**

The online version of this article (10.1186/s12940-019-0515-1) contains supplementary material, which is available to authorized users.

## Introduction

Environmental exposures play an important role in individual and population health. Traditionally, epidemiologists and toxicologists have focused on studying the toxicity of single environmental compounds and their health effects. However, every day we are simultaneously exposed to thousands of environmental contaminants which potentially interact and affect health differently as mixture components. More studies now focus on evaluating exposure to environmental mixtures, and new statistical methods are being adapted and developed for this task. These newer methods—some adapted from statistical machine learning and data science fields and some developed specifically for environmental mixtures—aim to overcome challenges that are incurred by more traditional biostatistical methods (e.g., high dimensionality, multi-collinearity, and multiple comparisons). Current and future methods should be employed with a specific research question in mind; as different methods exist to answer different questions, results from analyses using different methods are expected to vary.

In August 2018, the Columbia University Mailman School of Public Health Department of Environmental Health Sciences hosted a two-day Mixtures Workshop to introduce mixtures methods to environmental health science researchers [1]. During the Workshop, we—environmental epidemiologists and biostatisticians—came together to teach and discuss with a widely diverse scientific audience the major statistical methods currently used to study mixtures. The Workshop encompassed unsupervised methods such as clustering, principal component analysis (PCA), and exploratory factor analysis (EFA); and supervised methods such as variable selection (lasso, elastic net, and group lasso), weighted quantile sum (WQS) regression, and Bayesian kernel machine regression (BKMR). WQS and BKMR were specifically developed for environmental mixtures, while the other methods have been adapted from other fields. The Workshop focused on discussing each method’s statistical background, type of research question(s) it best addresses, and R packages available for its implementation. Our aim is to contribute to a better understanding of appropriate uses of mixture methods based on the research questions each method best answers.

To illustrate the methods discussed as part of this Workshop, it was important to use a publicly available real dataset (i.e., not simulated data) with high-dimensional environmental exposures, a biologically-relevant health outcome, and multiple plausible research questions. Given these criteria, we chose the paper by Mitro et al. that uses the 2001–2002 National Health and Nutrition Examination Survey (NHANES) dataset to investigate the association between exposure to persistent organic pollutants (POPs) with high affinity to the aryl hydrocarbon receptor (AhR) and longer leukocyte telomere length (LTL) [2]. The study’s results provide insight into a potential mechanism underlying poly-chlorinated biphenyl- (PCB-) and dioxin-related carcinogenesis mediated by activation of AhR and subsequent telomerase expression. However, Mitro et al. used potency-weighted sums, resulting in loss of information, which certain mixtures methods address [2]. Using the mixture methods previously listed, we tested the association between LTL and exposure to the same mixture of POPs analyzed by Mitro et al. [2]. We note, that although some of these methods have been used for prediction in other fields, our focus was not on prediction, but rather to understand how exposure to this mixture may impact LTL. In this paper, we describe the results we obtained using each method and how they compare to one another and to the results obtained by Mitro et al. [2].

Understanding the toxicity of environmental mixtures is pivotal for developing new policies and improved strategies to minimize toxic exposures. Thus, the Workshop’s overall objective, and consequently also the goal of this paper, is to expand the understanding of the appropriate use of existing methods to assess exposure to environmental mixtures by presenting a set of mixture methods as examples to address different research questions.

## Methods

### Study population

For our analyses, we used the same population used in the original paper by Mitro et al. [2]. Briefly, we used the 2001–2002 NHANES cycle, for which 11,039 people were interviewed. Of those over twenty years of age who provided blood samples and consented to DNA analysis, sufficient stored samples to estimate telomere length were available for 4,260 participants. From this population, we excluded individuals without environmental chemical analysis data (n = 2,850) or who were missing data on covariates (body mass index (BMI) (n = 70), education (n = 2), and serum cotinine (n = 8)). We further removed participants with any missing values for individual PCBs, dioxins, or furans (n = 327), resulting in a final study population of 1,003 participants, identical to the smallest sub-sample Mitro et al. included in the original analyses [2]. Participants provided written informed consent, and the institutional review board of the National Center for Health Statistics approved the survey [3].

### Exposure assessment

Exposure assessment of PCBs, dioxins, and furans has been described previously [2]. Briefly, congeners were isolated from serum samples using a C18 solid phase extraction. Analytical runs of all congeners were blinded and included blanks and quality control samples. All samples were measured using high-resolution gas chromatography/isotope-dilution high-resolution mass spectrometry [4, 5]. Limits of detection (LOD) were typically ∼2 ng/g and varied according to serum volume. The LOD range for each congener was reported by Mitro et al. [2]. Coefficients of variation differed by congener and sample lot [4, 5]. Congeners were adjusted for serum lipids which were calculated using an enzymatic summation method [6].

### Telomere length measurement

LTL measurement has been described previously [2]. Briefly, purified DNA was extracted from whole blood using the Puregene (D-50K) kit protocol (Gentra Systems, Inc., Minneapolis, MN) and stored at −80^∘^C. The quantitative polymerase chain reaction (qPCR) method was used to measure telomere length relative to standard reference DNA (T/S ratio) [7, 8]. Samples were assayed three times in duplicate wells, producing six data points which were averaged to calculate mean T/S ratios [9]. Analytical runs were blinded, and the CDC conducted a quality control review [2].

### Statistical analysis

All POP values were lipid-adjusted by the U.S. Centers for Disease Control and Prevention (CDC) [2, 4–9]. We included a total of eighteen congeners in all analyses: eight non–dioxin-like PCBs, two non–ortho-substituted PCBs, one mono–ortho-substituted PCB, three chlorinated dibenzo-p-dioxins, and four dibenzo-furans (see Additional file 1: Table S4 for the full list).

We excluded congeners whose concentrations were detected in fewer than 60% of samples [2]. For all remaining congeners that were included in our analyses with values below the LOD, we used the sample-specific LOD divided by the square root of two, as provided by the CDC [4, 5]. Single substitution of values below the LOD were performed to retain comparability with the original analysis [2].

We present the methods and results of our analyses in two groups: supervised and unsupervised methods. **Unsupervised** methods find a solution independently of any outcome(s) of interest, usually by reducing the dimensionality of the exposure matrix. This can be achieved by grouping exposures or by grouping observations (e.g., individuals in a cohort study), and is often performed as a preliminary or exploratory step. **Supervised** methods, conversely, allow the outcome of interest to inform the solution.

All unsupervised methods include the eighteen congeners of interest but do not include LTL to inform the solution nor control for covariates. The solution of these methods (clusters, components, or factors) were subsequently included in health models using LTL as the outcome and adjusting for all covariates included by Mitro et al. [2]: age, age^2^, sex, race/ethnicity (non-Hispanic white, non-Hispanic black, Mexican American, other), educational attainment (less than high school, high school graduate, some college, college or more), BMI (<25, 25–29.9, ≥ 30), serum cotinine, and blood cell count and distribution (white blood cell count, percent lymphocytes, percent monocytes, percent neutrophils, percent eosinophils, and percent basophils). All supervised methods include the eighteen congeners, LTL as the outcome, and adjust for the same covariates as listed above. POPs, LTL, and serum cotinine were log-transformed in all analyses to enhance comparability with the original study. While NHANES uses a probability-based sampling method to represent the non-institutionalized U.S. population [3], we did not include sampling weights in our analyses because our goal was to present these methods and not to obtain US-generalizable results. All analyses were conducted in R version 3.5.1, and all code, results, and graphs are available online at https://github.com/lizzyagibson/Mixtures.Workshop.2018. Additionally, available functions and packages in R and useful resources (i.e., articles and textbooks) are detailed in Additional file 1: Table S1.

#### Unsupervised methods

##### Clustering

is a dimensionality reduction technique used to identify distinct homogeneous subgroups in a given population [10, p. 385], i.e., the dataset is partitioned in a way that the most similar observations are grouped together. For mixtures analyses, clustering can be used to identify specific population subgroups that share a similar exposure profile. Although many different clustering methods exist, for this application we focused on the two most popular approaches: **k-means** and **hierarchical clustering** [10, p. 386].

For k-means, using the 18 log-transformed POPs, we evaluated the solutions of multiple cluster numbers (e.g., 2, 3, 5). By definition, as the number of clusters increases the within cluster variation will decrease; our goal, then, is to identify the smallest cluster number that sufficiently minimizes the within-cluster variation. We selected K = 3, since further increasing the cluster number minimally decreased the total within cluster variation. Expert knowledge, thus, is required in the selection of K and the interpretation of this, or any, clustering solution.

An alternative clustering approach to k-means is hierarchical clustering. The output of hierarchical clustering is a dendrogram, which is an upside-down tree-based representation of the clustered observations. At the bottom of the tree, the observations are represented by leaves which then fuse into branches moving up the tree. Early fusions (closer to the bottom) indicate more similarity among the observations relative to later fusions (higher up in the tree) [10, 11]. Various dissimilarity measures can be use to group observations in a dendogram [10], here we used complete linkage which uses the largest dissimilarity between the observations of two given clusters. Complete linkage tends to yield more balanced dendograms with defined clusters that are easier to visualize. A single tree can be used to obtain different numbers of clusters depending on the height at which it is cut. There is no metric to determine the best height to cut, and this process is subjectively based on visual analysis of the dengrogram. Here we cut the tree at a height of 11.25 to obtain three main clusters. We tried different heights to obtain two, three, and four clustered trees. The three clustered tree yielded the most balanced clusters; thus, we selected this solution for the health model analysis. Since there is not a consistently-used rule for selecting K or the height at which to cut a dendogram, it is important to try several options and consult experts on the topic at hand to better interpret the solution.

For both k-means and hierarchical clustering, we included cluster membership as a categorical exposure of interest in a multiple linear regression (controlling for covariates) to estimate the association between cluster membership and log-LTL.

##### Principal Component Analysis

(PCA) is another dimensionality reduction method based on the correlation matrix of the variables. In mixtures analyses, PCA can be used to identify common patterns in the exposure variables or observations; these can either represent common sources of the chemicals in the mixtures or common behaviors in the population. PCA aims to explain the total variance with fewer variables by creating new uncorrelated variables (principal components, PCs) based on linear combinations of the original variables. Each PC explains a percentage of the total variance with the first PC set to account for most of the total variance. Each subsequent component is determined as the one that explains most of the remaining variance and is orthogonal to (i.e., not correlated with) the previous components [10]. As PCA depends on variance to decompose a dataset, it is best to standardize the data first to make all chemical contributions equal and avoid allowing those with larger variances to disproportionately inform the solution. The PCA solution results in as many PCs as variables in the original matrix, making the choice of number of PCs to include in descriptive statistics or models subjective. One commonly used criterion is the percentage of variance explained (e.g., retain PCs sufficient to explain 80% of the variance). Another option is to visualize the results to determine where the decrease of explained variability by each added component levels off. In some applications, 65% of the total variance might be acceptable, while others might require >85%. Ultimately the choice will depend on the research question, overall objective, and interpretability of the solution. For the PCA analysis presented here, we a priori decided to select those PCs that accounted for ≥75% of the total variance. We then simultaneously included the PC scores (for PC1, PC2, and PC3) as continuous exposures in a multiple linear regression (controlling for covariates) to estimate the association with log-LTL.

##### Exploratory Factor Analysis

(EFA) assumes that the chemical concentrations arise from a specific number of unobserved (i.e., latent) factors. These latent factors represent common sources of variation among the exposure variables, accounting for their correlation structure. Uncorrelated error terms specific to each exposure variable account for the remaining unique variation [11]. EFA, thus, naturally lends itself to exposure pattern identification in mixtures analyses. Although EFA does not aim to explain the total variance in the data, like PCA, and instead aims to identify common sources of variation in the data, the solutions of both methods are often similar. The *u*^2^ estimates the “uniqueness” of the original variables, i.e., it measures the variance that is “unique” to the variable and not explained by the estimated factors. A high *u*^2^ implies that much of a variable’s variance is not explained by the factors. Due to EFA’s dependence on variance, it is also good to first standardize the dataset. Since the “true” originating mechanism is almost never known, neither model can provide the “correct” answer. Thus, both models should be used in conjunction with expert knowledge. For our analysis, we ran models with 2–5 factors with both orthogonal and oblique (i.e, correlated) rotations. We chose the model with the lowest empirically derived Bayesian Information Criterion (BIC) and the oblique rotation to enhance interpretability. Because environmental exposures are expected to be correlated, the oblique solution allows for more realistic results. As with the PCA results, we ran a health model to estimate the association between continuous factor scores (simultaneously included in the model) and log-LTL, adjusting for covariates.

#### Supervised methods

##### Variable Selection

determines which variables (here exposures) of a given set are most predictive of an outcome of interest to fit a single, more parsimonious regression model containing solely those variables [10]. Variable selection algorithms, thus, can be used in mixtures analyses to potentially identify the toxic agent(s) in the mixture. Variable selection is often used to improve prediction accuracy—with which environmental epidemiologists may not be concerned. However, in the presence of highly correlated exposures, these methods perform better than traditional regression methods and may provide a more interpretable solution [11]. Because a model that contains any subset of *p* candidate exposures has 2^*p*^ possible subsets of the total number of exposures (e.g., for this analysis *p* = 18; we would therefore need to consider 262,144 models, even before considering any interactions), trying every possible combination of exposures is infeasible and statistically unsound due to multiple comparisons. Instead, variable selection approaches provide an automated and efficient approach to better choose a smaller set of the exposure variables [10].

**Lasso** (least absolute shrinkage and selection operator) is a variable selection method that uses a penalty term to constrain the linear regression model with respect to the sum of the absolute values of the coefficients, shrinking some of them to zero [12]. The goal of lasso is to minimize the prediction error by keeping only those variables that are the most relevant to the outcome. The penalty term depends on a tuning parameter, *λ*: if *λ*=0, all variables are included in the model; as *λ*→*∞*, all variables are zeroed out [10, 11]. Here, we selected *λ* by minimizing the cross-validated prediction error. We only penalized the POP variables; the rest of the covariates were forced into the model to control for confounding bias. We then used the best *λ* value obtained from cross-validation to identify the POP variables with non-zero coefficients, that is, the variables that best predict the outcome (log-LTL). Before applying lasso, or any penalized method, it is important to standardize all predictors. This is because these methods include the coefficients—but not predictors (i.e. the exposures corresponding to the coefficients)—in their penalty terms, so the estimated coefficients depend on the scale of these exposures.

One drawback of lasso is that if the exposures are correlated (as they often are in environmental health applications), lasso will select a single variable from the correlated exposure matrix, increasing bias. **Elastic net** is a variable selection method, similar to lasso, that addresses this issue by encouraging grouping of correlated variables [10, 13]. Specifically, it allows coefficients of highly correlated variables to shrink towards each other instead of shrinking all except one entirely to zero. Elastic net achieves this by including a second penalty term, the sum of the squared coefficients, in addition to the sum of the absolute values [11, 13]. This requires a second tuning parameter, assigning *λ* a non-negative value over a grid of *α* values on [0,1] (in lasso, *α*=1 by default; if *α*=0, coefficients will approach, but will not reach zero) [14]. Here, we selected the best *α* and *λ* values based on minimization of the cross-validation prediction error, then used those values to identify the POP variables with non-zero coefficients.

With some prior information regarding groups of correlated exposures, another alternative to lasso is **group lasso**. For this method, the researcher needs to specify group membership for all exposures, and the penalty term is applied at the group level. In this way, all exposures within a group are either zeroed-out or not, and no single correlated exposure within an assigned group is separated from the rest. The penalty is a function of the number of exposures within each group, thus larger groups are penalized more [11]. We created three groups to correspond with the grouping used by Mitro et al. [2]: non–dioxin-like PCBs (eight PCBs with no toxic equivalency factor (TEF) and no AhR affinity: PCBs 74, 99, 138, 153, 170, 180, 187, and 194), non-ortho PCBs (two non–ortho-substituted PCBs with high TEFs and high AhR affinity: PCBs 126 and 169), and toxic equivalent POPs with moderate to high TEFs and AhR affinity(*m*ono-ortho-substituted *P*CB 118, four dibenzo-*f*urans, and three chlorinated dibenzo-p-*d*ioxins), here refer to as mPFD. We only penalized the POP variables (assigning all non-exposure covariates to a separate group that was forced in the model) and chose the best *λ* value by cross-validation to identify the POP variables with non-zero coefficients.

##### Weighted Quantile Sum Regression

(WQS) aims to assess the overall impact of a mixture on a specific outcome. It creates an empirically-weighted index of chemicals based on their quantiles and includes the index as a single exposure term in a regression model. The weighted index represents the overall mixture, and the chemical-specific weights are interpreted as relative variable importance levels using similarly scaled variables (e.g., quantiles; deciles were used herein), identifying potentially toxic agents [15–17]. WQS estimates variable weights using bootstrapped samples from a training set, then tests the effect of the weighted index in a separate test set [15]. A nonlinear numerical optimization algorithm estimates weights (constrained to be between 0 and 1 and to sum to 1) for each bootstrap sample in the training set. The final index is defined based on the weighted average for the weights across the bootstrap samples. For example, the average may be weighted based on the relative signal from each bootstrap sample (i.e., based on the test statistic for the beta coefficient for the index) [15, 18, 19]. The significance of the defined weighted quantile sum index is tested using the separate test set (generally, 60% of the sample size). We set an a priori cut-point for identifying important toxic agents (in case the coefficient for the index was significant) as a weight ≥1/*p*=1/18=0.06, i.e., weights that exceed the case of uniform weights. This cut-point is meant as a guideline; other weight aspects, such as variability and percent variance explained, should also be considered. WQS includes a further directionality constraint on the mixture members (but not the covariates), evaluating components in the direction of increased risk in an effort to improve interpretability of the weighted index [15]. For our analysis, we constrained the model in the positive direction.

##### Bayesian Kernel Machine Regression

(BKMR) is a semi-parametric technique, incorporating the benefits of both parametric (e.g., fewer parameters to estimate) and non-parametric (e.g., flexibility) methods into one model. BKMR flexibly models the combined effects of different chemicals, while allowing for nonlinear effects as well as interactions among them. Specifically, BKMR allows researchers to examine (1) whether the exposure to the mixture is associated with the outcome of interest; (2) the exposure-response relationships between individual chemical exposures and outcome; and (3) whether the components of the mixture interact. It includes the chemicals of interest in a non-parametric Gaussian kernel function within a Bayesian regression model [20–22]. The kernel does not impose a functional form on the exposure-response relationship, thus capturing a wide range of relationships, including nonlinear exposure-response functions and nonlinear and non-additive interactions among all mixture members [20, 23]. BKMR can perform either component-wise or hierarchical variable selection. Here, we employed hierarchical variable selection, which provides group importance scores (Posterior Inclusion Probabilities, PIPs) for pre-defined mutually-exclusive groups of variables, in addition to estimating the importance of a congener given that the group that contains that congener is important (conditional PIPs) [20–22]. For our analysis, we grouped the POPs into three groups—non-dioxin-like PCBs, non-ortho PCBs, and mPFD—as in the group lasso model. We additionally standardized all continuous variables (log-POPs, log-LTL, age, log-cotinine, and blood cell count and distribution) to improve computational efficiency.

## Results

**Fig. 1:**
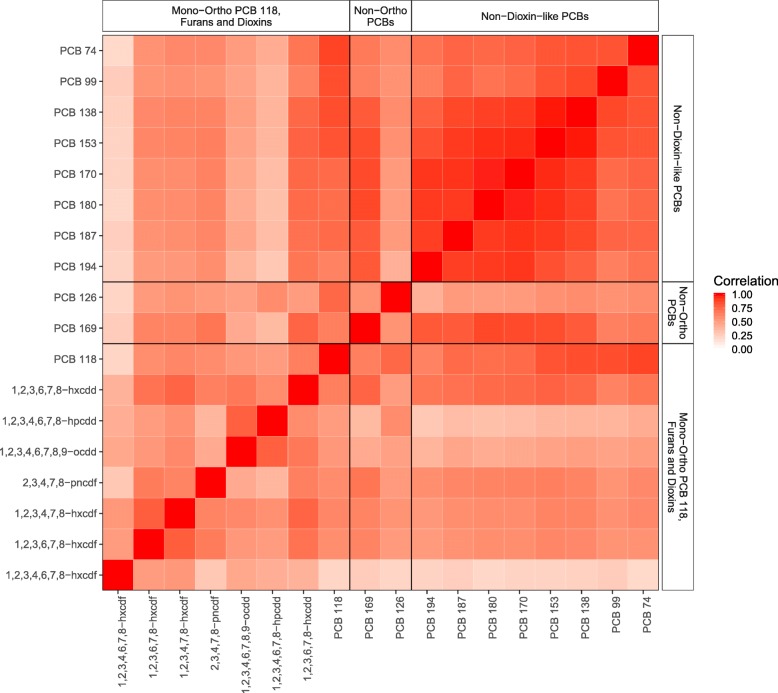
Correlation heatmap of lipid-adjusted POPs (*p* = 18) across participants in NHANES 2001–2002 (N = 1,003). Spearman correlation coefficients presented for untransformed distributions, sectioned according to groupings in the original [2] paper

Population demographics and congener concentration levels can be found in Additional file 1: Tables S4 and S5, respectively. POPs are moderately to highly correlated (Fig. 1). Spearman correlation coefficients within the non-dioxin-like PCBs are all ≥0.65 and as high as 0.98; correlations within the mPFD group range from 0.22 to 0.79; and the two non-ortho PCBs have a correlation of 0.55.

### Unsupervised methods

We present the results from all unsupervised methods in Table 1.

**Table 1:** Summary results of health models for the unsupervised methods: (K-means clustering, hierarchical clustering, PCA, and EFA)

Variable	*β*	95% CI	*P*-value
K-means clustering			
Cluster 1 (high exposure)	0.08	0.03, 0.13	0.001
Cluster 2 (medium exposure)	0.05	0.02, 0.09	0.005
Cluster 3 (low exposure)	*Reference*		—
Hierarchical clustering			
Cluster 1 (high exposure)	0.05	0.01, 0.10	0.03
Cluster 2 (medium exposure)	0.03	-0.00, 0.07	0.06
Cluster 3 (low exposure)	*Reference*		—
Principal Component Analysis			
PC1	-0.01	-0.02, -0.01	<0.001
PC2	0.001	-0.01, 0.01	0.87
PC3	0.002	-0.01, 0.02	0.76
Exploratory Factor Analysis			
FA1	-0.003	-0.03, 0.03	0.86
FA2	0.03	0.00, 0.05	0.02
FA3	0.03	0.01, 0.05	0.01
FA4	-0.02	-0.04, 0.00	0.06

#### Clustering

**Fig. 2:**
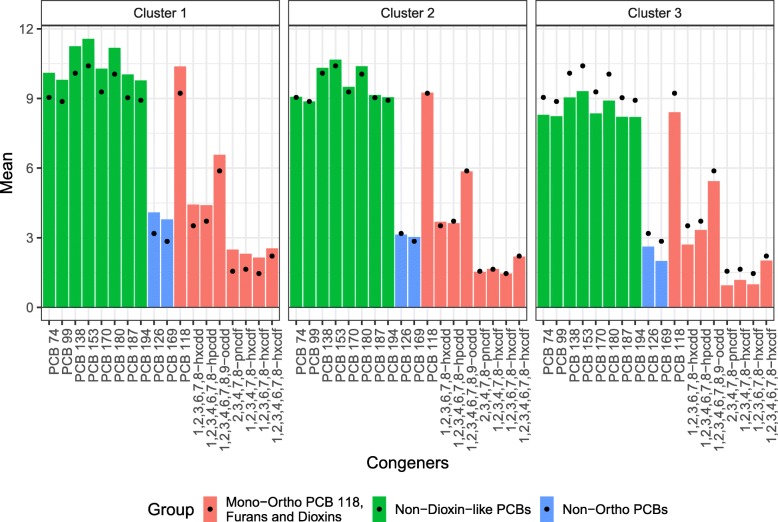
Clusters from k-means clustering. Mean level of POPs (*p* = 18) in three clusters of participants in NHANES 2001–2002 (N = 1,003). Points represent overall population average for each congener. Values are in log-transformed pg/g lipid. The color scheme represents the groupings from the original [2] paper

The clusters obtained by k-means and hierarchical clustering resulted in similar grouping patterns. In both methods, cluster 1 included participants with POP concentrations above the population average, cluster 2 included participants with POP concentrations close to the population average, and cluster 3 contained those participants with POP concentrations below the population average (Fig. 2).

Using cluster 3 as the reference (participants with POP concentrations below the population average), cluster 1 membership (participants with POP concentrations above the average) is significantly associated with longer log-LTL (*β*_k-means_ = 0.08, 95%CI = 0.03, 0.13; *β*_hierarchical_ = 0.05, 95%CI = 0.01, 0.10). This means that on average, members of k-means cluster 1 are expected to have longer log-LTL by 0.08 than members of cluster 3. Participants in cluster 2 (those with close to average exposures) have marginally longer log-LTL, on average, compared with the reference group (*β*_k-means_ = 0.05, 95%CI = 0.02, 0.09; *β*_hierarchical_ = 0.03, 95%CI = -0.002, 0.07). Among the non-POP variables, increasing age and the sex category male have a significantly negative association with telomere length in both models, as expected [24–26].

#### Principal component analysis

Using PCA, we identified three PCs which account for 79.7% of the total variability in POP exposure. The first PC had similar moderate variable loadings for all POPs in the same direction. The second PC separated the POPs by group: negative moderate loadings for all non-dioxin-like PCBs and non-ortho PCB 169, positive mainly high loadings for all mPFDs and non-ortho PCB 126. The third PC has high loadings for 1,2,3,4,6,7,8-hxcdf (mPFD), non-ortho PCB 126, and PCB 118 (mPFD) (Additional file 1: Figure S2). This means that PC1 scores decrease when all POPs increase; PC2 scores increase when mPFD congeners increase and non-dioxin-like PCBs decrease; and PC3 scores increase when PCB 126, and PCB 118 increase and 1,2,3,4,6,7,8-hxcdf decreases.

PC2 (*β*_PC2_ = 0.001, 95%CI = -0.01, 0.01) and PC3 (*β*_PC3_ = 0.002, 95%CI = -0.01, 0.02) were not associated with log-LTL. PC1 (*β*_PC1_ = -0.01, 95%CI = -0.02, -0.01) was significantly negatively associated with log-LTL. Per one unit increase in PC1 score, log-LTL decreases by 0.01; since all POPs have negative loadings on PC1, this means that an increase in all POPs is associated, on average, with a 0.01 increase in log-LTL. As in the clustering methods, increasing age and the sex category male are also negatively associated with the outcome.

#### Exploratory factor analysis

We chose the four-factor model based on its empirically derived BIC and the oblique rotation to enhance interpretability. The first factor had high variable loadings on most non-dioxin-like PCBs (PCBs 138, 153, 170, 180, 187, and 194), one non-ortho PCB (PCB 169), and one dioxin (1,2,3,6,7,8-hxcdd). The second factor had high loadings on all four furans. The third factor had high loadings on two non-dioxin-like PCBs (PCBs 74 and 99), one mPFD (PCB 118), and one non-ortho PCB (PCB 126). The fourth factor had high loadings on two dioxins (1,2,3,4,6,7,8-hpcdd, and 1,2,3,4,6,7,8,9-ocdd) (Additional file 1: Figure S3). The factors were moderately (r = 0.38 between factors one and four) to highly (r = 0.74 between factors one and three) correlated. Together, these factors explained 79.0% of the common variability. Furan 1,2,3,4,6,7,8 hxcdf and PCB 126 were the most unique (*u*^2^= 0.63 and 0.51, respectively), indicating that most of the variance in these two variables is not shared with other variables in the overall factor model. We included individual factor scores in the health model as four continuous variables, controlling for covariates. Factor one (*β*_F1_= -0.003, 95%CI = -0.03, 0.03) was not associated with log-LTL. Factor four (*β*_F4_= -0.02, 95%CI = -0.04, 0.001) was negatively, but non-significantly associated with log-LTL. Factors two (*β*_F2_= 0.03, 95%CI = 0.004, 0.05) and three (*β*_F3_= 0.03, 95%CI = 0.01, 0.05) were significantly positively associated with log-LTL. This means that a one-unit increase in Factor 2 score was associated, on average, with a 0.03 increase in log-LTL. Increasing age and male sex were, again, negatively associated with log-LTL.

### Supervised methods

#### Variable selection

**Fig. 3:**
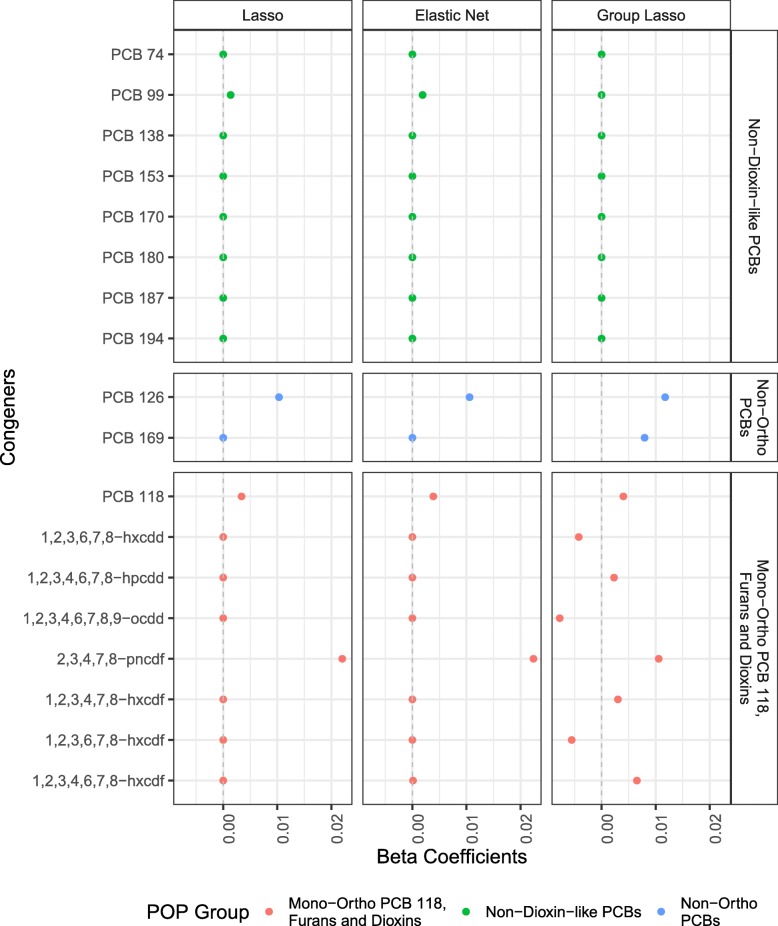
Coefficients for POPs (*p* = 18) from variable selection models. Models adjusted for age, age^2^, sex, race/ethnicity, educational attainment, BMI, serum cotinine, and blood cell count and distribution. All POP concentrations (pg/g) were log-transformed and standardized. The color scheme represents the groupings from the original [2] paper

For **lasso**, the best *λ* value obtained from cross-validation is 0.0034. Using this *λ* in the lasso model generated non-zero coefficients for four of the 18 POP variables (Fig. 3): PCB 99, a non-dioxin-like PCB with no TEF and no AhR affinity (*β*= 0.001), PCB 118, included in mPFD (*β*= 0.003), furan 2,3,4,7,8-pncdf (*β*= 0.02, i.e., a one standard deviation increase in furan 2,3,4,7,8-pncdf is associated with a 0.02 unit increase in log-LTL), and PCB 126, a non-ortho PCB (*β*= 0.013).

With **elastic net**, we estimated *α*= 0.8 and *λ*= 0.0039 from cross-validation, similar to the *λ* chosen for the lasso model. The elastic net model using these *λ* and *α* values generated non-zero coefficients for five of the 18 POP variables (Fig. 3). The same four variables selected by lasso (PCB 99 (*β*= 0.002), PCB 118 (*β*= 0.004), furan 2,3,4,7,8-pncdf (*β*= 0.02, i.e., a one standard deviation increase in PCB 126 is associated with a 0.01 unit increase in log-LTL), and PCB 126 (*β*= 0.01), plus furan 1,2,3,4,6,7,8 hxcdf (*β*= 0.0001). Overall, the lasso and elastic net models selected almost the same congeners and estimated similar variable coefficients, which is not surprising given the high *α* value and very similar *λ* values.

Based on cross-validation, we chose a *λ* value of 0.006 for the **group lasso** model. Group lasso pushed the eight coefficients for non-dioxin-like PCBs to zero, with non-zero coefficients for the two non-ortho PCBs and the eight mPFDs (Fig. 3). Increases in both non-ortho PCBs predicted longer log-LTL (PCB 126 *β*= 0.01, PCB 169 *β*= 0.008). This means that a one standard deviation increase in PCB 169 is associated with a 0.01 unit increase in log-LTL. Increases in three furans (2,3,4,7,8-pncdf *β* = 0.01, 1,2,3,4,7,8-hxcdf *β*= 0.003, 1,2,3,4,6,7,8-hxcdf *β*= 0.006) were associated with longer log-LTL, while increased furan 1,2,3,6,7,8-hxcdf (*β*= -0.005) was associated with shorter log-LTL. Increased exposure to dioxin 1,2,3,4,6,7,8-hpcdd (*β*= 0.002) was associated with longer log-LTL, but the two other dioxins (1,2,3,6,7,8-hxcdd (*β*= -0.004), 1,2,3,4,6,7,8,9-ocdd (*β*= -0.008)) were associated with shorter log-LTL. PCB 118 (*β*= 0.004) was associated with longer log-LTL. Increasing age and male sex were negatively associated with log-LTL in all variable selection models.

#### Weighted quantile sum regression

**Fig. 4:**
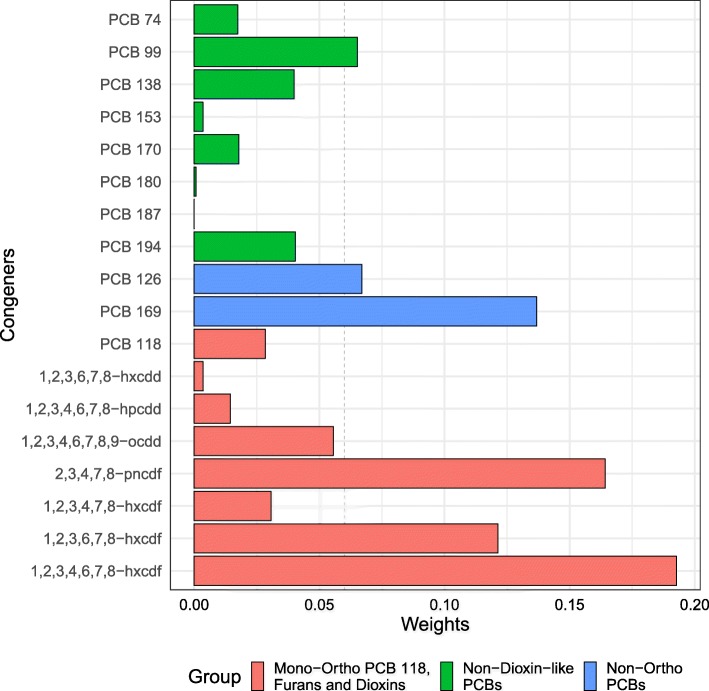
Variable weights from the WQS index. Barplot shows weights assigned to each congener. Model adjusted for age, age^2^, sex, race/ethnicity, educational attainment, BMI, serum cotinine, and blood cell count and distribution. The color scheme represents the groupings from the original [2] paper. The dashed line at 0.06 indicates the cut-point for identifying potentially toxic agents

The coefficient for the mixture index in the WQS model was positive (*β*= 0.02, 95%CI = 0.01, 0.03). As the overall mixture effect was statistically significant, individual POP weights can be interpreted as variable importance factors. Six POPs had weights ≥0.06 (Fig. 4), three furans (1,2,3,4,6,7,8-hxcdf weight = 0.19, 2,3,4,7,8-pncdf weight = 0.16, and 1,2,3,6,7,8-hxcdf weight = 0.12), both non-ortho PCB (PCB 169 weight = 0.14 and PCB 126 weight = 0.07), and one non-dioxin-like PCB (PCB 99 weight = 0.06). These congeners were the largest contributors to the mixture effect, with the first six congeners explaining 75% of the total weights. In the WQS model, increasing age and male sex were negatively associated with log-LTL.

#### Bayesian kernel machine regression

In the BKMR model, the mPFD group had the highest PIP (=0.87), making it the most important group in the mixture. The next most important group was the non-ortho PCBs (PIP = 0.62), followed by non-dioxin-like PCBs (PIP = 0.42). Within the mPFD group, furan 2,3,4,7,8-pncdf contributed the most to the model (conditional PIP = 0.88). The next most important POP in the mPFD group, PCB 118, had a conditional PIP of 0.06. In the non-ortho PCB group, PCB 126 contributed more (conditional PIP = 0.65) than PCB 169 (conditional PIP = 0.35). Within the non-dioxin-like PCBs, PCB 170 had the highest PIP (conditional PIP = 0.17).

**Fig. 5:**
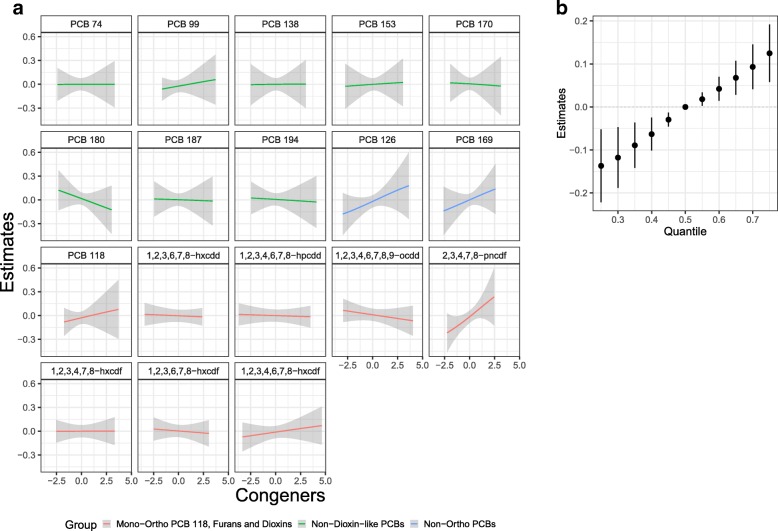
**a** Congener-specific effect estimates of mixture members on log-LTL in NHANES 2001–2002 participants estimated by BKMR. Single congener associations and 95% credible bands are presented with other POPs fixed at their median. **b** Overall effect of the mixture on log-LTL (estimates and 95% credible intervals), comparing log-LTL when all exposures are at a particular quantile to the median. The model adjusted for age, age^2^, sex, race/ethnicity, educational attainment, BMI, serum cotinine, and blood cell count and distribution. All congener concentrations (pg/g) were log-transformed and standardized

The independent congener associations all appear relatively linear (Fig. 5). One furan (2,3,4,7,8-pncdf) was statistically significantly associated with log-LTL, and there was suggestive evidence of positive associations with PCBs 126 and 169. Other congeners also had positive associations (PCBs 99, 118, and furan 1,2,3,4,6,7,8-hxcdf); some appeared to have negative associations (PCB 180 and dioxin 1,2,3,4,6,7,8,9-ocdd), and many had no association (PCBs 74, 138, 153, 170, 187, and 194, two dioxins, 1,2,3,6,7,8-hxcdd and 1,2,3,4,6,7,8-hpcdd, and two furans, 1,2,3,4,7,8-hxcdf and 1,2,3,6,7,8-hxcdf). We observed no interaction among mixture members (Additional file 1: Figures S4 and S5), but we did find a significant overall mixture effect, with higher exposure to the mixture associated with longer log-LTL (Fig. 5). Parameter estimates for increasing age and male sex were negative.

## Discussion

Using the methods presented here as a toolbox, we demonstrate the range of potential research questions that can be addressed using a single dataset, as well as the importance of defining the research question and selecting the appropriate method for analysis a priori (Table 2). We aim to show that results across methods—though not always comparable—can be complementary and, in our example, show a degree of consistency. Unsupervised methods all addressed research questions pertaining to dimensionality reduction and pattern recognition, which can help identify exposure sources or shared behaviors. Since clusters, PCs, and factors are not based on a health outcome, they will be the same regardless of the health outcome being considered. Supervised methods answer research questions concerning associations with a specific health outcome and can help identify potentially toxic agents in a mixture, synergistic activities among a mixture’s components, or estimate the overall mixture effect on a specific health outcome. In this case study, similar conclusions can be drawn from unsupervised and supervised approaches: an overall mixture effect is found using multiple methods, and the same individual congeners are identified as toxic across analyses. However, it is important to note that such consistent interpretation of the various results is not always guaranteed, and expert knowledge is required to interpret the solutions of both supervised and unsupervised methods.

**Table 2:** Summary of methods, research questions best answered, and findings

Method	Research Question	Results
Unsupervised Methods		
K-means Clustering Hierarchical Clustering	Are there population subgroups that share similar exposure profiles?	The study population is clustered by level of exposure: high, average, and low. High exposure is associated with longer log-LTL.
PCA	Are there specific patterns in POP exposure?	Three patterns of exposure were identified. Exposure to all POPs (PC1) is associated with longer log-LTL.
EFA		Four patterns of exposure were identified. Exposure to all four furans (FA2) and to PCBs 118 and 126 (FA3) is associated with longer log-LTL.
Supervised Methods		
Lasso	Which congeners are associated with changes in log-LTL?	PCB 99, PCB 118, PCB 126, and furan 2,3,4,7,8-pncdf are associated with longer log-LTL.
Elastic Net		PCB 99, PCB 118, PCB 126, furan 2,3,4,7,8-pncdf, and furan 1,2,3,4,6,7,8 hxcdf are associated with longer log-LTL.
Group Lasso	Which a priori defined congener groups are associated with changes in log-LTL and what is the magnitude of the association with congeners within those groups?	All mPFD congeners are associated with log-LTL, with variability in direction—five mPFDs with longer log-LTL, and three mPFDs with shorter log-LTL. Non-ortho PCBs (PCBs 126 and 169) are associated with longer log-LTL.
WQS	What is the overall effect of the mixture on log-LTL? What congeners are most important?	The mixture index is associated with longer log-LTL. Three furans and both non-ortho PCBs are important mixture members. Furans 1,2,3,4,6,7,8-hxcdf and 2,3,4, 7,8-pncdf has the largest weights.
BKMR	Is there an association between the mixture and log-LTL? What is the exposure-response relationship between each congener and log-LTL? Are there interactions between congeners?	The overall mixture is associated with longer log-LTL. Furan 2,3,4,7,8-pncdf, PCB 126, and PCB 169 are independently associated with longer log-LTL. No interactions or nonlinearities were found.

All 18 congeners were included in all unsupervised and supervised models

Beginning with unsupervised methods, we applied (1) clustering to group individuals by similar exposure profiles, which can help identify shared behaviors; and (2) PCA and EFA to identify the major sources of variation in the data, which can answer questions regarding exposure patterns. Both k-means and hierarchical clustering grouped individuals by high, medium, or low exposure across POPs, indicating a significantly positive overall mixture effect. PCA identified three components that explained the majority of the variation in the data: a component with moderate loadings from all POPs, a component that separates non-dioxin-like PCBs from mPFD congeners, and a third more mixed component. EFA identified four underlying sources of common variation, grouping POPs generally by their respective classes as non-dioxin-like PCBs, furans, dioxins, and a mixture of those remaining. Among the supervised methods, variable selection methods can help identify potentially toxic agents in a mixture. All three methods employed here (lasso, elastic net, and group lasso) identified PCB 126, PCB 118, and furan 2,3,4,7,8-pncdf as those congeners most strongly associated with increased log-LTL. WQS aims to quantify the overall effect of a mixture and the importance of mixture members. With WQS, we found a significantly positive overall mixture effect and identified furans 1,2,3,4,6,7,8-hxcdf, 2,3,4,7,8-pncdf, and 1,2,3,6,7,8-hxcdf, as well as PCBs 169, 126, and 99 as potentially toxic agents. BKMR answers research questions about independent effects of mixture members, interactions among them, and the overall mixture effect. BKMR identified furan 2,3,4,7,8-pncdf, PCB 126, and PCB 169 as the most toxic congeners in the mixture and found a significantly positive overall mixture effect, but no nonlinearities or interactions.

It is usually not easy to draw conclusions across results from models used to answer different research questions, as results are not comparable. However, we were able to identify some reassuring consistencies across analyses. This is likely the case because in these particular data the associations all appear linear with no evidence of interactions, so the assumptions from all the implemented models are reasonable. In other cases in which this is not true, there will likely be greater differences in results across models, and the exploration of these differences, together with the knowledge of what each approach assumes, can point the user to identify features in the exposure-response relationship that might not be discovered otherwise.

Older age and male sex were negatively associated with log-LTL across all analyses, as expected [24–26]. In pattern identification, the resulting health models followed the directionality expected based on Mitro et al.’s findings [2]. Health models including clusters estimated associations between higher exposures and log-LTL; health models including PCs estimated an association between the general POP exposure PC and log-LTL; and health models including factors estimated associations between log-LTL and factors consisting of furans, mono-ortho PCBs, and non-ortho PCBs. Variable selection models, WQS, and BKMR all identified furan 2,3,4,7,8-pncdf and PCB 126 as potentially toxic agents. Group lasso, WQS, and BKMR all identified PCB 169 as potentially toxic, as well. WQS and BKMR both estimated a significantly positive overall mixture effect, an interpretation that could also be drawn from the clustering results, in this specific analysis.

Though we included a continuous outcome measure in these analyses, all methods discussed can accommodate different outcome distributions and study designs. Unsupervised methods may be used in a two-stage analysis with any health model of choice. Variable selection models can also be implemented on binary, categorical, count, and time-to-event data, and can also accommodate clustering in the outcomes, e.g., repeated measures. WQS and BKMR have both been extended to included binary outcomes, with more extensions in progress, such as for survival analyses [27].

Please note that, from a toxicity perspective, mono-ortho PCB 118 should stand alone, instead of being grouped with furans and dioxins [28]. This, however, would complicate comparison with the original Mitro et al. paper [2]. A priori grouping only affects results from group lasso and hierarchical BKMR—for biological correctness, we changed the groupings in these two models, separating PCB 118 into its own group. The BKMR results did not noticeably change (results not shown). The group lasso results were equivalent in direction and magnitude, with the exception that the PCB 118 coefficient was much larger in the 4-group solution (results not shown). This is expected as smaller groups receive a smaller penalty [11]. Additionally, we used the sample-specific LOD divided by the square root of two for values below the LOD to enhance comparability with the original analysis. Mitro et al. performed sensitivity analyses with multiple imputations examining the effects of the treatment of data below the LOD on the overall results and found that single substitution did not meaningfully alter the findings [2].

While our results are generally consistent with those of Mitro et al. [2], their study addressed a different research question. They used expert knowledge to group POPs into three categories: non-dioxin-like PCBs with no TEFs, non-ortho PCBs with high TEFs and high AhR affinity, and mPFD congeners which include furans and dioxins with high TEFs and high AhR affinity, and one mono-ortho PCB. With these a priori defined groups, their analysis did not intend to identify specific toxic agents, interactions, or quantify the overall effect of the mixture. Mitro et al. found that the non-dioxin-like PCB group, when controlling for non-ortho PCBs, had no effect on log-LTL [2]. Our lasso, elastic net, and WQS models identified the non-dioxin-like PCB 99 (out of eight) as a potentially toxic agent, but when grouped together in group lasso and BKMR, associations were null. Mitro et al. also found significant associations between mPFD and non-ortho PCB groups and log-LTL [2]. Their analysis did not aim to select toxic agents from these groups, but our analyses identified some members of these groups as toxic agents. This comparison highlights differences between biologically- and data-driven approaches. Mitro et al. weighted non-ortho PCBs and mPFD congeners by their respective TEFs, creating a weighted index [2]. This allowed for a biologically meaningful grouping strategy that is not easy or always feasible to replicate with other mixtures, as most chemicals do not have a TEF analog. When not much is known about the chemicals in a mixture, data-driven methods are necessary to address the statistical issues inherent in mixtures. However, when more is known about a mixture, comparing data-driven to biologically-driven approaches and incorporating biological information into data-driven approaches can yield more interpretable and reproducible results.

To the best of our knowledge, a couple of papers have been published comparing different methods for environmental mixtures, one assessing *in utero* phthalate exposure and birth weight, and one on exposure to metals and cardiovascular disease [29, 30]. Although these are significant contributions to the field of mixtures methods in environmental health, they do not focus on describing why different approaches are more appropriate for different research questions, nor when each method should be used [31].

Each method discussed, however, comes with its own limitations. All unsupervised methods are somewhat subjective. While there are “guidelines” and indices for choosing the right number of clusters, PCs, or factors, the ultimate decision relies on expert knowledge and interpretable results, and different researchers are bound to make different choices. Including cluster membership, PC scores, or factor scores in the health model (as we did, here) ignores the uncertainty inherent in the solution selection, resulting in underestimated confidence intervals and, potentially, spurious results [32]. Clustering, further, reduces a high-dimensional exposure matrix to a single categorical variable, resulting in a substantial loss of information. Both PCA and EFA assume linear combinations of variables and do not allow for potential interactions between mixture members in the pattern identification. PCA solutions, specifically, are not easily interpretable—even with expert knowledge, PCs are not always intelligible—and the orthogonal solution is often not realistic for environmental mixtures. For variable selection, the inability to estimate confidence intervals as a means of gauging uncertainty in the estimates is a considerable limitation. To address this, some researchers have implemented a two-step process, inserting the variables selected from a penalization method into a traditional regression model. This will yield invalid inferences, as the uncertainty of the first step is not propagated into the second, and the coefficients from penalized methods are unlikely to equal those from traditional regression—they may even be in opposite directions [12]. Furthermore, the dependence of the estimated coefficients on the scaling of the predictors, and—in the case of the group lasso—on the size of the group, may greatly hinder interpretability of these estimates. WQS categorizes continuous variables into quantiles to reduce the impact of extreme concentrations; however, the quantiles reduce the amount of information in the exposure matrix. The effects of all mixture members must be in the same direction, and no corresponding effect estimates are given for potentially toxic agents, only variable importance weights. Neither WQS nor variable selection methods allow for interactions, unless hard-coded by the researcher. And WQS’s reliance on both training and testing sets further requires a sufficiently large sample size. BKMR demands a large sample size, as well, because of the non-parametric kernel function. Instead of reducing the problem to a few coefficients, the kernel estimates the smooth shape of the mixture-response relationship, resulting in less power for a given sample size. Furthermore, this process is quite computationally expensive compared with the other methods discussed here. A table detailing the advantages, disadvantages, assumptions, and required outcome distributions for each method is included in Additional file 1: Table S2.

Moreover, though these methods address many of the traditional regression short-comings, they fail to overcome limitations that are shared by all environmental mixtures methods. For the incorporation of missing data, these methods—like traditional regression—require complete case analysis, but all—even the most computationally intensive—can be combined with a multiple imputation procedure to allow for uncertainty concerning missingness. Especially when biomarkers are used, the timing of sample collection with respect to exposure—and potentially critical windows of exposure—and the half-lives of analytes must be considered. All supervised approaches are at risk of selecting a chemical with high concentrations during both the critical window of exposure and sampling which co-occurred with the toxic agent. If the actual toxic agent has a short half-life, it may be below the LOD at sampling or have an excessively noisy measurement depending on the variation in time between the critical window and sampling. Given varying measurement error across and high correlation between mixture members, any statistical method will choose a chemical measured with less error than the toxic agent, as long as the two are correlated [33, 34]. It is even possible that the mixture of interest does not include the actual toxic agent(s), or that the toxic agent does not provide an adequate number of measurements above the LOD. In all these cases, focusing on toxic agent(s) will lead to wrong conclusions and incorrect identification, regardless of the method. Mixtures including only a small number of mixture members may amplify these issues due to residual confounding from correlated, unmeasured co-pollutants, but even in larger mixtures, correlated exposure variables may amplify rather than reduce confounding bias from shared sources [35]. Finally, the mixture in this example was only comprised of lipophilic species, but combinations of lipophilic and hydrophilic species may result in enhanced and more pronounced toxicity, especially at low concentrations.

## Conclusion

Throughout the Workshop and this paper, we stressed the importance of choosing the correct mixtures method to answer a specific research question, as the results of different methods are interpreted quite differently. Thus, the aim of this Workshop and paper was to instruct environmental health researchers in methods to address several distinct types of mixtures questions, when to use these methods, and how to implement them. The results of the methods employed are not directly comparable, but they are generally consistent with each other and with the more biologically-driven results of Mitro et al. in identifying the mixture, or individual mixture members, as toxic [2]. To-date, and to the best of our knowledge, no single method exists to answer all mixtures questions, but with a well-defined research question and modern mixture methods, researchers are better equipped to explore complex relationships between environmental mixtures and adverse health outcomes.

## Additional file

Additional file 1 Supplemental description of methods and additional tables and figures. **Table S1** Useful resources for included methods. **Table S2** Overview of method characteristics. **Table S3** POP characteristics. **Table S4** Demographic Characteristics. **Figure S1** Hierarchical clustering dendrogram. **Figure S2** PCA loadings. **Figure S3** EFA loadings. **Figure S4** BKMR Interactions (Plot 1). **Figure S5** BKMR Interactions (Plot 2). (PDF 1233 kb)

## Data Availability

The datasets generated and/or analysed during the current study are available in the Data repository, https://github.com/lizzyagibson/Mixtures.Workshop.2018.
