# Supplementary material for: An overview of methods to address distinct research questions on environmental mixtures: an application to persistent organic pollutants and leukocyte telomere length
Source: Environ Health. 2019 Aug 28;18:76. doi: 10.1186/s12940-019-0515-1 (PMC6714427; doi:10.1186/s12940-019-0515-1)
Supplement: Supplementary file 1 — Supplemental description of methods and additional tables and figures. Table S1 Useful resources for included methods. Table S2 Overview of method characteristics. Table S3 POP characteristics. Table S4 Demographic Characteristics. Figure S1 Hierarchical clustering dendrogram. Figure S2 PCA loadings. Figure S3 EFA loadings. Figure S4 BKMR Interactions (Plot 1). Figure S5 BKMR Interactions (Plot 2). (PDF 1233 kb) [file 12940_2019_515_MOESM1_ESM.pdf]

Supplemental Material

# An Overview of Methods to Address Distinct Research Questions on Environmental Mixtures: An Application to Persistent Organic Pollutants and Leukocyte Telomere Length

Elizabeth A. Gibson, Yanelli Nunez, Ahlam Abuawad, Ami R. Zota, Stefano Renzetti, Katrina L. Devick, Chris Gennings, Jeff Goldsmith, Brent A. Coull, Marianthi-Anna Kioumourtzoglou

## List of Tables

SM.1 Useful resources for included methods. . . . . . . . . . . . . . . . . . . . . . . . . . . 2

SM.2 Overview of method characteristics. . . . . . . . . . . . . . . . . . . . . . . . . . . 3

SM.2 Overview of method characteristics (continued). . . . . . . . . . . . . . . . . . . . . 4

SM.3 POP characteristics . . . . . . . . . . . . . . . . . . . . . . . . . . . . . . . . . . 5

SM.4 Demographic Characteristics . . . . . . . . . . . . . . . . . . . . . . . . . . . . . 6

## List of Figures

SM.1 Hierarchical clustering dendrogram . . . . . . . . . . . . . . . . . . . . . . . . . . 7

SM.2 PCA loadings . . . . . . . . . . . . . . . . . . . . . . . . . . . . . . . . . . . . . 8

SM.3 EFA loadings . . . . . . . . . . . . . . . . . . . . . . . . . . . . . . . . . . . . . 9

SM.4 BKMR Interactions (Plot 1) . . . . . . . . . . . . . . . . . . . . . . . . . . . . . 10

SM.5 BKMR Interactions (Plot 2) . . . . . . . . . . . . . . . . . . . . . . . . . . . . . 11

**Table SM.1:** Useful resources for included methods.

| Method | R Functions and Packages[†] | Resources |
|---|---|---|
| Unsupervised Methods | | |
| K-means Clustering | `kmeans() in stats` | James et al., ch. 10.3.1 (2013), Friedman et al., ch. 14.3.6 (2001), Bishop, ch. 9.1 (2006) |
| Hierarchical Clustering | `hclust() in stats` | James et al., ch. 10.3.2 (2013), Friedman et al., ch. 14.3.12 (2001) |
| PCA | `prcomp() and princomp() in stats` | James et al., ch. 10.2 (2013), Friedman et al., ch. 14.5.1 (2001) |
| EFA | `factanal() in stats; fa() in psych` | Bartholomew et al. (2011), Pett et al. (2003), Friedman et al., ch. 14.7.1 (2001) |
| Supervised Methods | | |
| Lasso | `glmnet() in glmnet` | Tibshirani (1996), James et al., ch. 6.2.2 (2013), Friedman et al., ch. 3.4.2 (2001) |
| Elastic Net | `glmnet() in glmnet` | Zou and Hastie (2005), Friedman et al., ch. 3.4.3 (2001) |
| Group Lasso | `grpreg() in grpreg` | Yuan and Lin (2006), Friedman et al., ch. 3.8.4 (2001) |
| WQS | `gwqs() in gWQS` | Gennings et al. (2013), Carrico et al. (2015), Gennings et al. (2010) |
| BKMR | `kmbayes() in bkmr` | Bobb et al. (2018), Bobb et al. (2014) |

[†]Not meant to be a comprehensive list of available packages; includes packages used in this analysis and popular alternatives.

**Table SM.2:** Overview of method characteristics.

| Method | Pros | Cons | Assumptions† | Outcome Distributions |
|---|---|---|---|---|
| | | Unsupervised Methods | | |
| K-means Clustering<br><br>Hierarchical Clustering | Reduces dimensionality; clusters possibly interpretable | No gold standard for solution selection, user selects solution based on prior knowledge (i.e., implicit assumptions); susceptible to local instead of global minimum | Continuous exposures | NA |
| PCA | Reduces dimensionality | No gold standard for solution selection, user selects solution based on prior knowledge (i.e., implicit assumptions); interpretability not guaranteed; scores and components centered at zero | Continuous exposures | NA |
| EFA | Reduces dimensionality; factors allowed to be correlated; possibly interpretable | No gold standard for solution selection, user selects solution based on prior knowledge (i.e., implicit assumptions); scores and factors centered at zero | Continuous exposures | NA |
| | | Supervised Methods | | |
| Lasso | Sparse solution (i.e., unimportant variable coefficients pushed to zero) | No p-values or confidence intervals; arbitrary member of correlated variable coefficients pushed to zero | Linear exposure-response; errors are normally distributed and have constant variance (for continuous outcomes) | Gaussian (continuous), binomial (yes/no), Poisson (counts), multinomial (categorical), Cox (time-to-event), and clustered observations (e.g., repeated measures) |

**Table SM.2:** Overview of method characteristics (continued).

| Method | Pros | Cons | Assumptions† | Outcome Distributions |
|---|---|---|---|---|
| | | Supervised Methods | | |
| Elastic Net | Sparse solution (i.e., unimportant variable coefficients pushed to zero); correlated variable coefficients pushed toward each other | No p-values or confidence intervals | Linear exposure-response; errors are normally distributed and have constant variance (for continuous outcomes) | Gaussian (continuous), binomial (yes/no), Poisson (counts), multinomial (categorical), Cox (time-to-event), clustered observations (e.g., repeated measures) |
| Group Lasso | Sparse solution (i.e., unimportant variable coefficients pushed to zero); incorporation of known group membership | No p-values or confidence intervals; group size affects coefficient penalty | Linear exposure-response; errors are normally distributed and have constant variance (for continuous outcomes) | Gaussian (continuous), binomial (yes/no), Poisson (counts) |
| WQS | Parameterization means more statistical power; Ability to identify potentially toxic agents and overall mixture effect | No beta coefficients for individual mixture members; lose information with quantiles | Linear exposure-response; errors are normally distributed and have constant variance (for continuous outcomes); directionality constraints | Gaussian (continuous), binomial (logistic; yes/no), extensions in progress |
| BKMR | Ability to identify non-linear effects, interactions, and overall mixture effect; flexible | No p-values; computationally intensive (i.e., slow) | Prior distributions, hyperparameters, and tuning parameters | Gaussian (continuous), binomial (probit; yes/no), extensions in progress |

†All methods assume missing values are missing completely at random (MCAR). Some software may require users to remove missing values prior to implementation.

**Table SM.3:** Lipid-adjusted serum congener levels and LODs in NHANES 2001–2002 (N = 1,003). Concentrations are presented as pg/g lipid, unless otherwise noted.

| Congener | % > LOD | Mean | St Dev | 25% | Median | 75% |
|---|---|---|---|---|---|---|
| Non-Dioxin-like PBCs | | | | | | |
| PCB 74† | 67.5 | 12.6 | 13.6 | 4.1 | 7.4 | 15.6 |
| PCB 99† | 61.9 | 10.0 | 10.4 | 3.7 | 6.3 | 11.7 |
| PCB 138† | 93.4 | 38.1 | 40.4 | 11.7 | 25.7 | 50.0 |
| PCB 153† | 96.4 | 52.2 | 53.5 | 16.1 | 36.2 | 68.4 |
| PCB 170† | 74.9 | 15.5 | 14.4 | 4.9 | 11.4 | 21.7 |
| PCB 180† | 88.9 | 37.9 | 39.6 | 10.2 | 27.4 | 53.5 |
| PCB 187† | 65.9 | 12.1 | 12.3 | 4.0 | 8.0 | 16.1 |
| PCB 194† | 61.0 | 10.5 | 10.8 | 3.7 | 6.9 | 14.4 |
| Non-ortho PCBs | | | | | | |
| PCB 126 | 88.8 | 38.6 | 52.8 | 14.9 | 24.8 | 43.5 |
| PCB 169 | 88.3 | 24.5 | 20.6 | 9.4 | 18.5 | 34.6 |
| Mono-ortho PCB | | | | | | |
| PCB 118† | 73.8 | 16.7 | 23.3 | 4.5 | 9.5 | 19.5 |
| Dioxins | | | | | | |
| 1,2,3,6,7,8-hxcdd | 92.9 | 48.1 | 40.4 | 21.4 | 38.0 | 63.0 |
| 1,2,3,4,6,7,8-hpcdd | 98.9 | 57.4 | 55.4 | 23.9 | 41.7 | 72.3 |
| 1,2,3,4,6,7,8,9-ocdd | 80.7 | 494.1 | 518.2 | 196.9 | 346.0 | 600.0 |
| Furans | | | | | | |
| 2,3,4,7,8-pncdf | 65.3 | 6.7 | 5.7 | 2.3 | 5.2 | 9.2 |
| 1,2,3,4,7,8-hxcdf | 81.0 | 6.4 | 4.9 | 3.2 | 5.3 | 7.9 |
| 1,2,3,6,7,8-hxcdf | 69.2 | 5.4 | 4.2 | 2.6 | 4.3 | 6.9 |
| 1,2,3,4,6,7,8-hxcd | 89.4 | 11.6 | 10.6 | 6.4 | 9.8 | 14.1 |

†These concentrations are presented in ng/g lipid.

**Table SM.4:** Demographic characteristics in NHANES 2001–2002 (N = 1,003).

| Variable | Participants N | (%) |
|---|---|---|
| Age (years) | | |
| 20–39 | 383 | 38.2 |
| 40–59 | 312 | 31.1 |
| $\geq$ 60 | 307 | 30.6 |
| BMI (kg/m$^2$) | | |
| < 25 | 316 | 31.5 |
| 25–29.9 | 373 | 37.2 |
| $\geq$ 30 | 313 | 31.2 |
| Serum cotinine (ng/mL) | | |
| < 0.015 | 220 | 22.0 |
| 0.015–9.90 | 527 | 52.6 |
| $\geq$ 9.91 | 255 | 25.4 |
| Education | | |
| < High School | 317 | 31.6 |
| High school graduate | 228 | 22.8 |
| Some college | 269 | 26.8 |
| $\geq$ College graduate | 188 | 18.8 |
| Sex | | |
| Female | 558 | 55.7 |
| Male | 444 | 44.3 |
| Race/ethnicity | | |
| Other | 78 | 7.8 |
| Mexican American | 226 | 22.6 |
| Non-Hispanic black | 180 | 18.0 |
| Non-Hispanic white | 518 | 51.7 |

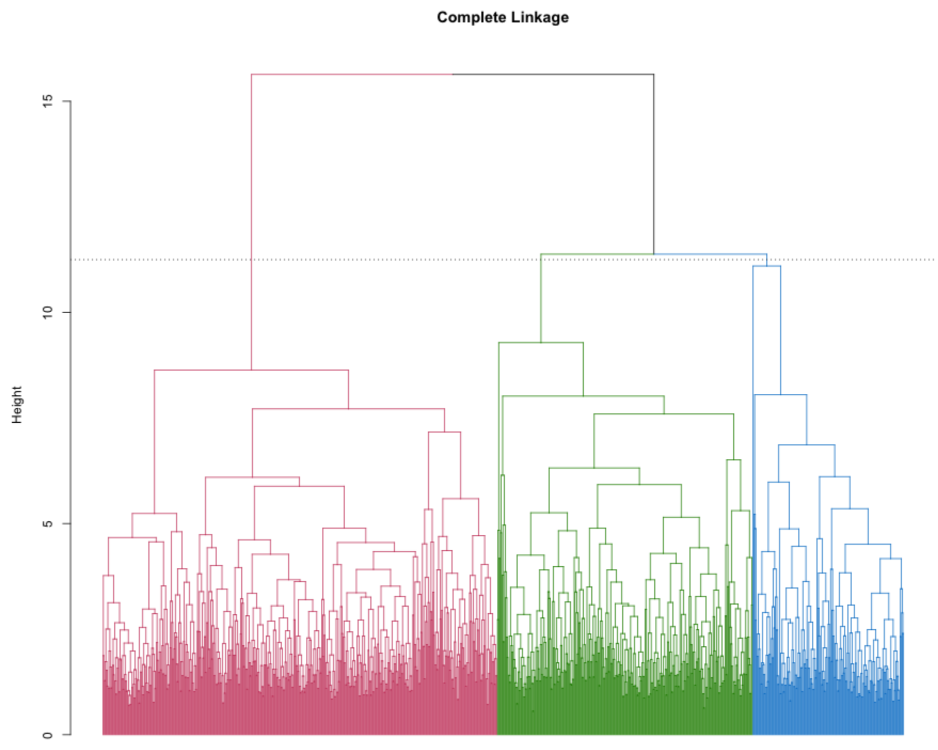


**Figure SM.1:** Hierarchical clustering dendrogram using complete linkage among POPs in NHANES 2001–2002 participants (N = 1,003). Complete linkage is one of many dissimilarity measures that tends to produce more compact clusters. It computes all pairwise dissimilarities between the participants in cluster one and the participants in clusters two, and considers the maximum value of these dissimilarities as the distance between the two clusters.

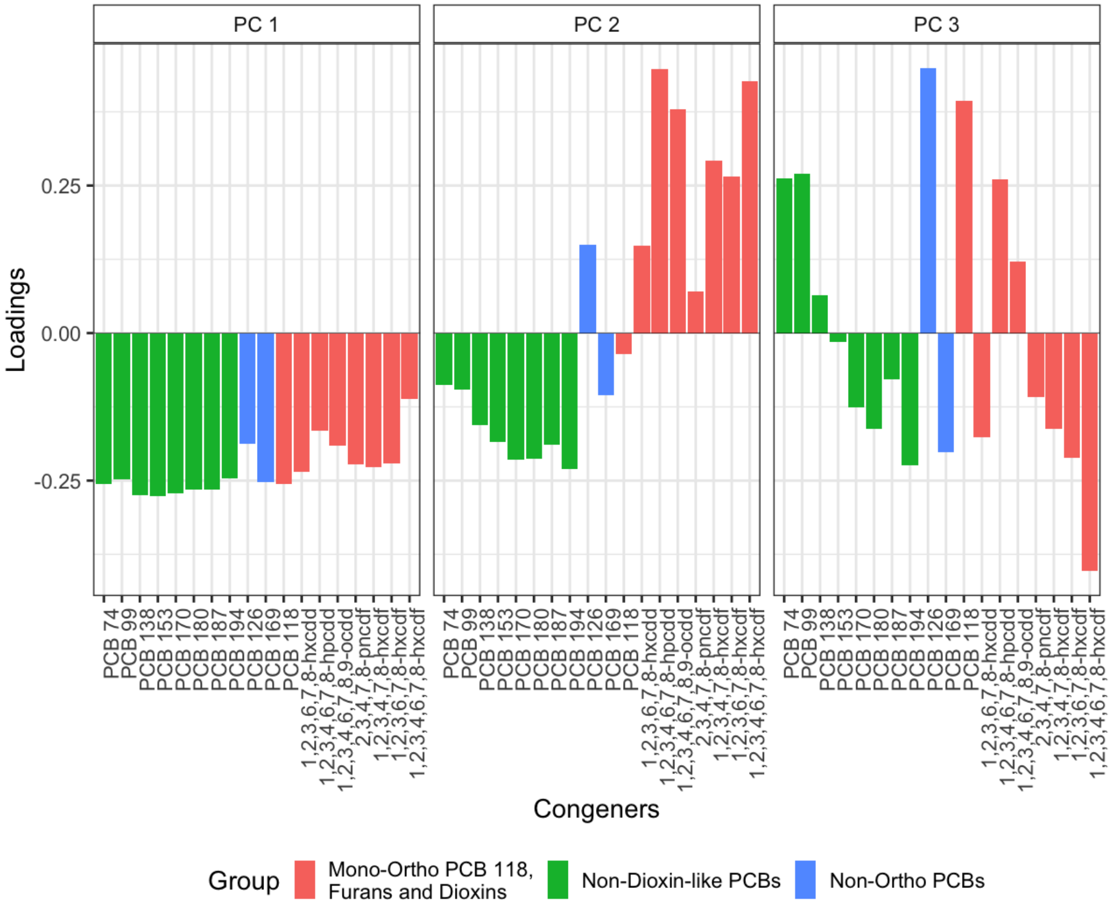


**Figure SM.2:** Individual congener loadings for the first three principle components in PCA. All POP concentrations (pg/g) were log-transformed and standardized. The coloring scheme represents the groupings from the original Mitro et al. (2015) paper.

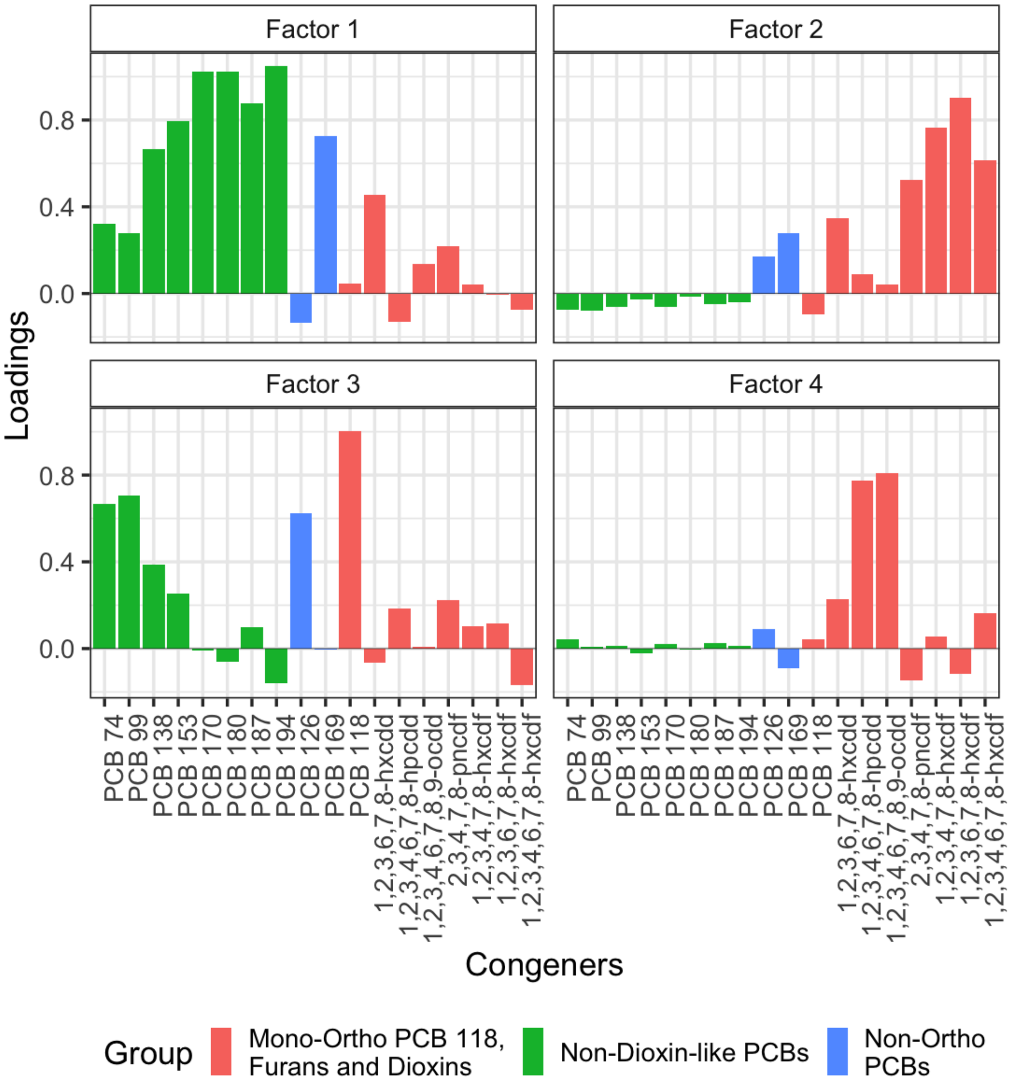


**Figure SM.3:** Individual congener loadings for the four factors in EFA. All POP concentrations (pg/g) were log-transformed and standardized. The coloring scheme represents the groupings from the original Mitro et al. (2015) paper.

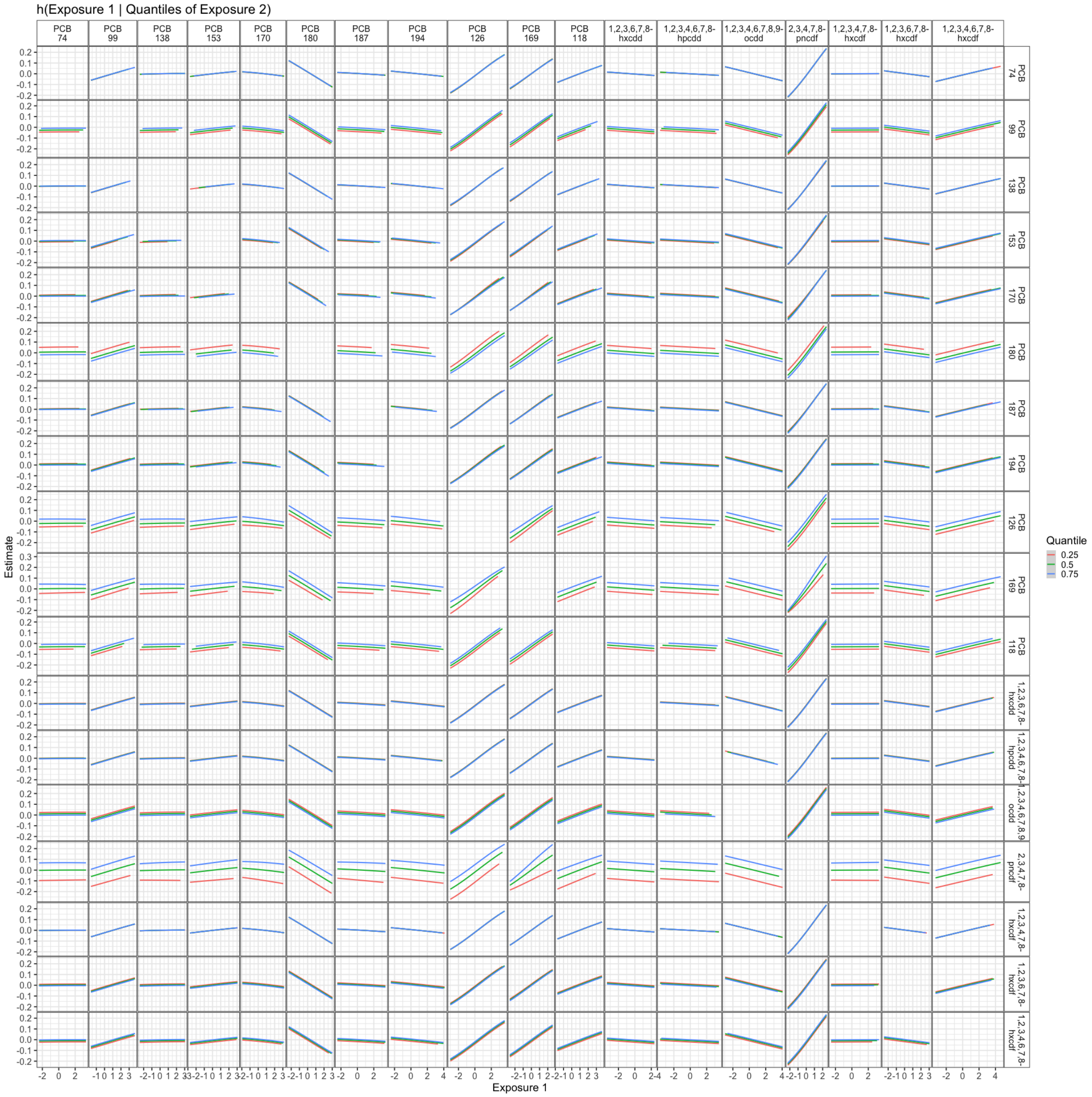


**Figure SM.4:** Congener-specific effect estimates of mixture members on LTL in NHANES 2001–2002 participants at varying levels of other POP estimated by BKMR. Bivariate exposure-response functions for POPs in columns when the row POP is fixed at either the $25^{\text{th}}$, $50^{\text{th}}$, or $75^{\text{th}}$ percentile and the remaining POPs are fixed at their medians. Model adjusted for age, age$^2$, sex, race/ethnicity, educational attainment, BMI, serum cotinine, and blood cell count and distribution. All POP concentrations (pg/g) were log-transformed and standardized.

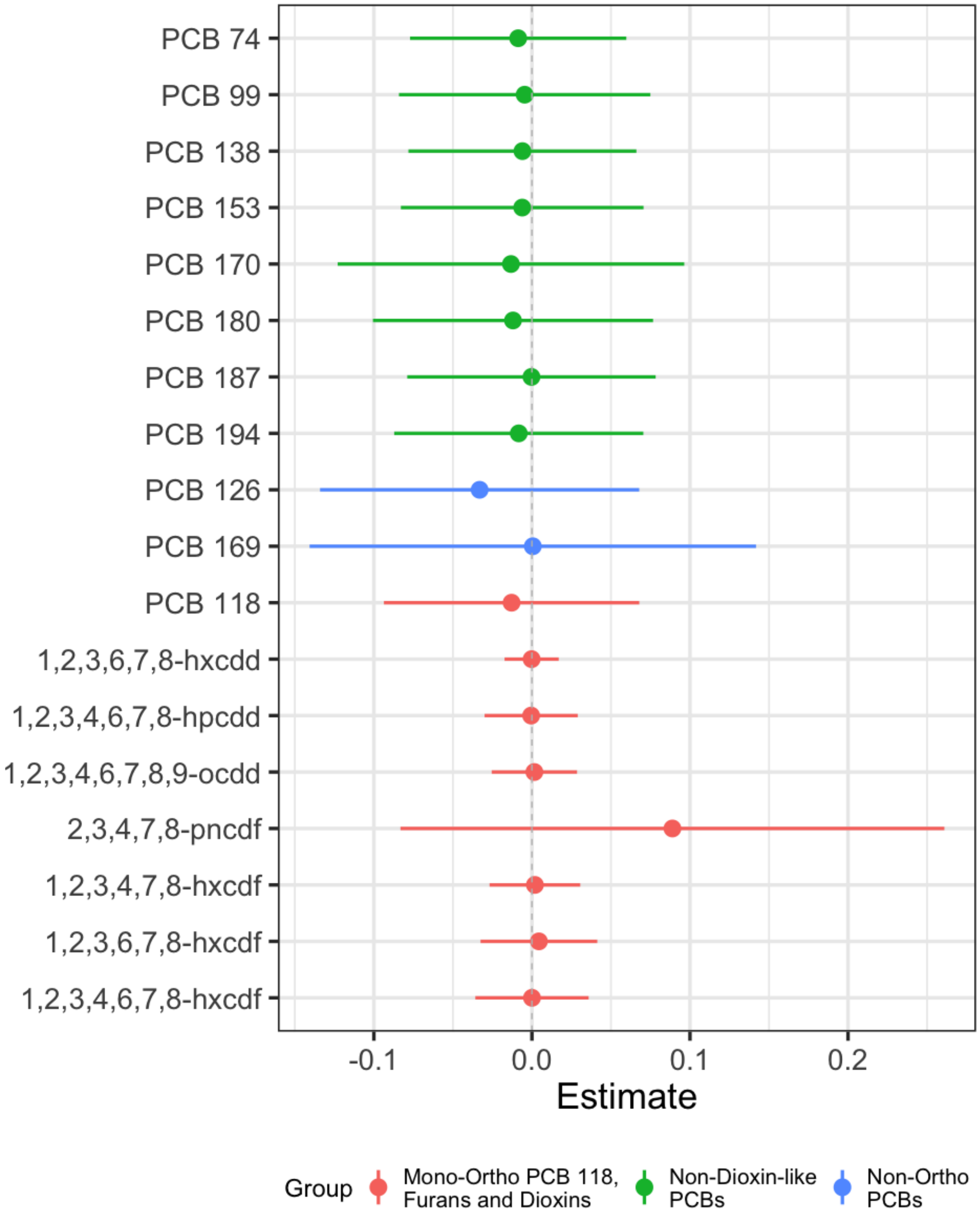


**Figure SM.5:** Interaction terms for individual mixture members and the remaining POPs in NHANES 2001–2002 participants estimated by BKMR. Each point represents the difference between the effect size of the congener when all other POPs are held at their 75$^{th}$ percentiles and the effect size of the same congener when all other POPs are held at their 25$^{th}$ percentiles. Range indicates 95% credible interval. Model adjusted for age, age$^2$, sex, race/ethnicity, educational attainment, BMI, serum cotinine, and blood cell count and distribution. All POP concentrations (pg/g) were log-transformed and standardized.

## References

Mitro, S. D., Birnbaum, L. S., Needham, B. L., and Zota, A. R. (2015). Cross-sectional associations between exposure to persistent organic pollutants and leukocyte telomere length among us adults in nhanes, 2001–2002. *Environmental health perspectives*, 124(5):651–658.
